# Metabolic engineering of *Escherichia coli* for efficient biosynthesis of fluorescent phycobiliprotein

**DOI:** 10.1186/s12934-019-1100-6

**Published:** 2019-03-20

**Authors:** Huaxin Chen, Peng Jiang

**Affiliations:** 1 Key Laboratory of Experimental Marine Biology, Institute of Oceanology, Chinese Academy of Sciences, Qingdao, 266071 China; 2 Laboratory for Marine Biology and Biotechnology, Qingdao National Laboratory for Marine Science and Technology, Qingdao, 266071 China; 3 Center for Ocean Mega-Science, Chinese Academy of Sciences, Qingdao, 266071 China

**Keywords:** Phycobiliprotein, Phycobilin, Chromophorylation ratio, Plasmid stability, *E. coli*

## Abstract

**Background:**

Phycobiliproteins (PBPs) are light-harvesting protein found in cyanobacteria, red algae and the cryptomonads. They have been widely used as fluorescent labels in cytometry and immunofluorescence analysis. A number of PBPs has been produced in metabolically engineered *Escherichia coli*. However, the recombinant PBPs are incompletely chromophorylated, and the underlying mechanisms are not clear.

**Results and discussion:**

In this work, a pathway for SLA-PEB [a fusion protein of streptavidin and allophycocyanin that covalently binds phycoerythrobilin (PEB)] biosynthesis in *E. coli* was constructed using a single-expression plasmid strategy. Compared with a previous *E. coli* strain transformed with dual plasmids, the *E. coli* strain transformed with a single plasmid showed increased plasmid stability and produced SLA-PEB with a higher chromophorylation ratio. To achieve full chromophorylation of SLA-PEB, directed evolution was employed to improve the catalytic performance of lyase CpcS. In addition, the catalytic abilities of heme oxygenases from different cyanobacteria were investigated based on biliverdin IXα and PEB accumulation. Upregulation of the heme biosynthetic pathway genes was also carried out to increase heme availability and PEB biosynthesis in *E. coli*. Fed-batch fermentation was conducted for the strain V5ALD, which produced recombinant SLA-PEB with a chromophorylation ratio of 96.7%.

**Conclusion:**

In addition to reporting the highest chromophorylation ratio of recombinant PBPs to date, this work demonstrated strategies for improving the chromophorylation of recombinant protein, especially biliprotein with heme, or its derivatives as a prosthetic group.

**Electronic supplementary material:**

The online version of this article (10.1186/s12934-019-1100-6) contains supplementary material, which is available to authorized users.

## Background

The phycobiliproteins (PBPs) are a family of light-harvesting proteins found in cyanobacteria, red algae and the cryptomonads [1]. Based on their spectrum properties, PBPs are classified into three main types: phycoerythrin (PE), phycocyanin (PC), and allophycocyanin (APC). These proteins absorb strongly in the visible region of the spectrum because they carry various covalently attached linear tetrapyrrole prosthetic groups (phycobilins). Due to their excellent fluorescent properties and their ability to be covalently linked to various biomolecules, PBPs have been widely used as fluorescent labels in flow cytometry and immunofluorescence analysis [2].

PBPs are generally extracted and purified from cyanobacteria and red algae [3]. When used as a fluorescent label, phycobiliprotein is often chemically cross-linked to streptavidin [4]. Streptavidin, a biotin-binding protein, is composed of four molecules of streptavidin monomer with four active sites for biotin binding [5]. The affinity of streptavidin for biotin is extremely high, and the Kd value is up to 10^−13^ [6]. The streptavidin-linked phycobiliprotein can bind to biotinylated proteins, DNA or other biochemical reagents and can thus be used to probe biological events.

In recent decades, pathways for the most abundant PBPs in cyanobacteria, APC (ApcA/ApcB) and PC (CpcA/CpcB), have been elucidated. Phycocyanobilin (PCB) and phycoerythrobilin (PEB) are the major phycobilins attached to APC and PC. These phycobilins originate from cyclic tetrapyrrole (heme). The oxidative cleavage of heme by heme oxygenase (Ho1) produces biliverdin IXα (BV), the first intermediate common to all phycobilins. BV is subsequently reduced by ferredoxin-dependent bilin reductases to yield PCB, PEB or other types of phycobilins [7]. The attachment of phycobilins to apo-PBPs is catalyzed by different type of lyases [8]. The CpcE/F lyase is the first identified enzyme dedicated to phycobilin attachment. This lyase has broad substrate specificity and catalyzes the attachment of PCB to the PC α subunit. The second identified lyase, CpcS/U, is responsible for the attachment of PCB to the Cys-82 residues on the APC α and β subunits and on the PC β subunit. In some cyanobacteria, such as *Synechococcus* sp. PCC 7002 and *Synechocystis* sp. PCC 6803, CpcS/U is composed of a heterodimer of two similar proteins, designated CpcS-I and CpcU [9, 10], while in other cyanobacteria, such as *Nostoc* sp. PCC 7120, the CpcS lyase exists in a monomeric, single subunit form [11]. The third identified CpcT lyase is a homodimer and catalyzes the attachment of PCB to the Cys-153 residue in the PC β subunit [12]. With the development of metabolic engineering techniques, biosynthetic pathways for PBPs have been constructed, and a number of holo-PBPs had been successfully produced in *Escherichia coli* [13–16]. These recombinant PBPs could easily be purified using immobilized metal affinity chromatography, and they retained the spectroscopic properties of native phycobiliprotein. In our recent work, the biosynthetic pathway for production of SLA-PEB (a fusion protein of streptavidin and allophycocyanin that covalently binds PEB) was constructed in *E. coli*. The fusion protein with PEB as chromophore was successfully produced in *E. coli*. While retaining the fluorescent properties, the fusion protein could bind biotinynated antibody and could be used as fluorescent label in immunofluorescence assay [17].

The recombinant PBPs produced in *E. coli*, however, were partially chromophorylated. The chromophorylation ratio of PBPs (the percentage of chromophorylated PBP to total recombinant PBP) was reported to range from 17.4 to 71.9% [14, 17, 18], indicating that a faction of the recombinant PBP was produced as its apo-form in *E. coli*. The apo-PBPs are difficult to remove from the PBP mixture during protein purification and lead to low detection sensitivity in immunofluorescence assays due to the lack of fluorescence emission. It was speculated that the incomplete chromophorylation is due to unfavorable codon usage that limits the expression of large amounts of lyases and phycobilins by *E. coli,* or it is due to aggregation of the recombinant proteins into insoluble inclusion bodies [13]. In addition, since heme serves as the precursor for phycobilin biosynthesis, depletion of heme could be a potential limiting factor for the chromophorylation of recombinant PBPs. In a recent work, an in vitro chromophore attachment reaction system was established in an attempt to improve the chromophorylation of recombinant PBP [19]. Spectral analysis showed that phycobilin attached rapidly to the recombinant PBP during the reaction. The chromophorylation ratio was elevated from 21.1 to 86.5%. Immunofluorescence assays showed that recombinant PBP with a higher chromophorylation ratio had a higher detection signal. However, the in vitro chromophore attachment reaction is time- and labor-consuming due to the need to purify phycobilin, lyases and recombinant PBPs.

In this study, we tried to elucidate the mechanisms underlying the incomplete chromophorylation of recombinant SLA-PEB and construct an efficient pathway for SLA-PEB production in *E. coli*. It was found that the *E. coli* strain transformed with dual expression plasmids lost one or both plasmids during the course of fermentation. To improve plasmid stability, we reconstructed the biosynthetic pathway in *E.* coli by using one expression vector. This strain, which has increased plasmid stability, retained the entire SLA-PEB biosynthetic pathway and produced recombinant SLA-PEB with a higher chromophorylation ratio. To achieve full chromophorylation, the catalytic abilities of Ho1 from different cyanobacteria were investigated based on BV and PEB accumulation. Upregulation of the heme biosynthetic pathway genes was also carried out to increase heme availability and PEB biosynthesis in *E. coli*. These strategies enable the efficient production of SLA-PEB in *E. coli*. In a 5 L fermentor, the engineered strain V5ALD produced 56.4 mg/L SLA-PEB with a chromophorylation ratio of 96.7%.

## Materials and methods

### Construction of plasmids and strains

**Fig. 1:**
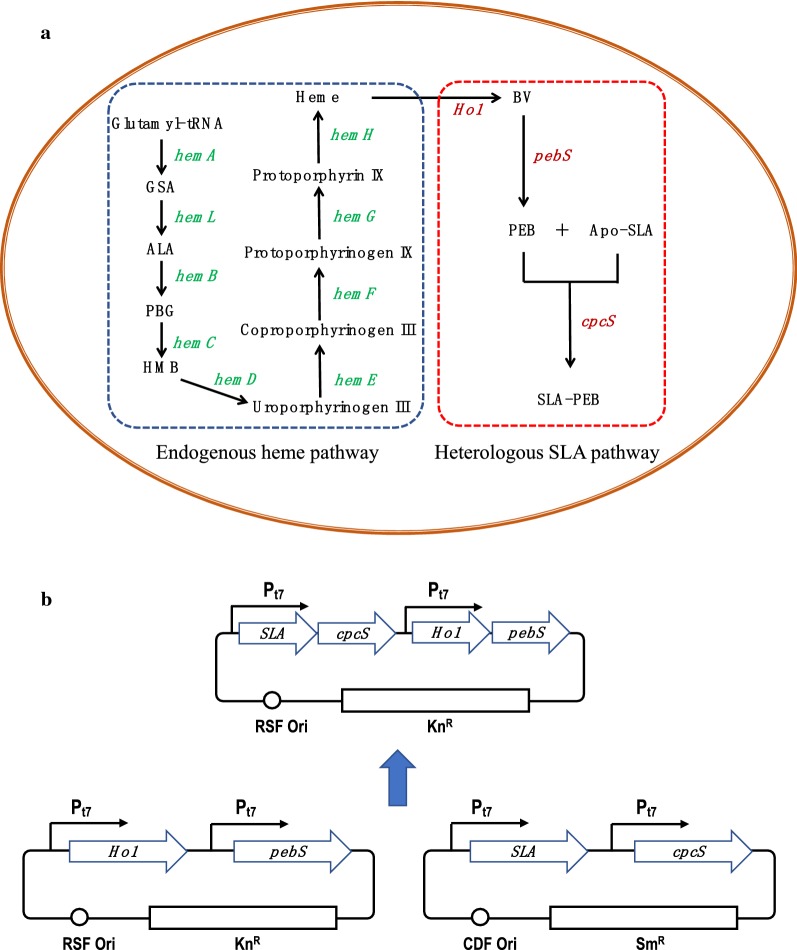
Schematic presentation of strategy for SLA-PEB biosynthesis in *E. coli*. **a** Pathway for SLA-PEB biosynthesis. The pathway for SLA-PEB is divided into two modules: the endogenous heme pathway and the heterologous SLA pathway. *GSA* glutamate-1-semialdehyde, *ALA* 5-aminolevulinic acid, *PBG* porphobilinogen, *HMB* hydroxymethylbilane, *HemA* glutamyl-tRNA reductase, *HemL* glutamate-1-semialdehyde aminotransferase, *HemB* 5-aminolevulinic acid dehydratase, *HemC* porphobilinogen deaminase, *HemD* uroporphyrinogen III synthase, *HemE* uroporphyrinogen decarboxylase, *HemF* coproporphyrinogen III oxidase, *HemG* protoporphyrin oxidase, *HemH* ferrochelatase, *Ho1* heme oxygenase 1, *pebS* phycoerythrobilin synthase, *CpcS* lyase. **b** Plasmid construction: the four genes required for SLA-PEB biosynthesis were cloned into the plasmids pCDFDuet-1 and pRSFDuet-1 or into a single plasmid pRSFDuet-1

**Fig. 2:**
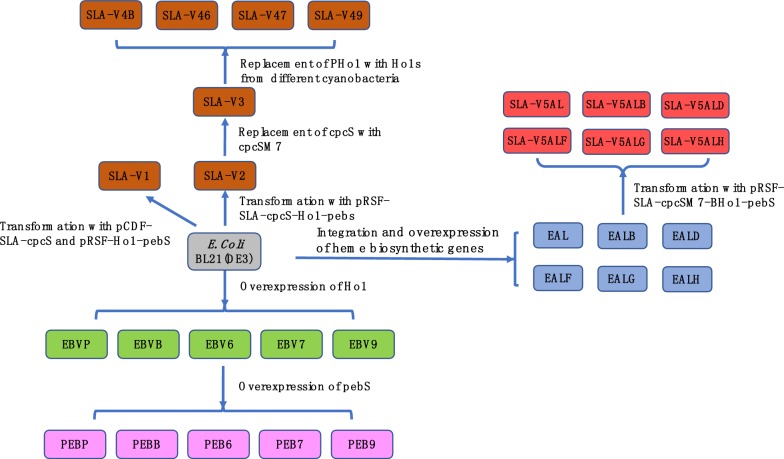
Flow diagram of the strain construction in this study

An *E. coli* strain for SLA-PEB biosynthesis was constructed in our previous work [17]. This engineered strain (denoted hereafter as SLA-V1) contained two expression plasmids: pCDF-*SLA*-*cpcS* and pRSF-*PHo1*-*pebS*. The fusion protein SLA-PEB with 6× His tag at its N-terminal can be easily purified by Ni^2+^ chelating affinity chromatography. To express the SLA-PEB pathway genes from one expression vector, *PHo1* and *pebS* (coding phycoerythrobilin synthase) were designed as a polycistron. This polycistron was chemically synthesized and ligated into the second expression cassette of the vector pRSFDuet-1, generating the plasmid pRSF-*PHo1*-*pebS*. The *Bam*HI/*Sac*I fragment of *SLA* from pCDF-*SLA*-*cpcS* was ligated into similarly digested pRSF-*PHo1*-*pebS*, generating the plasmid pRSF-*SLA*-*PHo1*-*pebS*. *cpcS* was amplified by PCR using primers cpcSF and cpcSR with a ribosomal binding site engineered on the primer cpcSF. The PCR product was digested with *Sac*I/*Sal*I, purified and ligated into similarly digested pRSF-*SLA*-*PHo1*-*pebS*, generating the expression plasmid pRSF-*SLA*-*cpcS*-*PHo1*-*pebS* (Fig. 1). This plasmid was then transformed into competent *E. coli* BL21(DE3), generating the strain SLA-V2 (Fig. 2).

*Ho1* genes from *Synechococcus* sp. PCC 7002(*7Ho1*), *Synechococcus elongatus* BP-1(*BHo1*), *Synechococcus* sp. PCC 9311(*9Ho1*), *Synechocystis* sp. PCC 6803(*6Ho1*) and cyanophage PSSM-2(*PHo1*) were optimized according to *E. coli* codon usage and artificially synthesized. These *Ho1* genes were digested with *Nde*I*/Bgl*II and cloned into similarly digested pRSFDuet-1, generating the plasmids pRSF-*7Ho1*, pRSF-*BHo1*, pRSF-*9Ho1*, pRSF-*6Ho1* and pRSF-*PHo1*. These plasmids were individually transformed into *E. coli* BL21(DE3), generating the BV-producing strains EBV7, EBVB, EBV9, EBV6 and EBVP. The *pebS* gene was amplified using the primers pebSF and pebSR, digested with *Bgl*II*/Kpn*I and ligated into similarly digested pRSF-*7Ho1*, pRSF-*BHo1*, pRSF-*9Ho1*, pRSF-*6Ho1* and pRSF-*PHo1*, generating the plasmids pRSF-*7Ho1*-*pebS*, pRSF-*BHo1*-*pebS*, pRSF-*9Ho1*-*pebS*, pRSF-*6Ho1*-*pebS* and pRSF-*PHo1*-*pebS*, respectively. These plasmids were individually transformed into competent *E. coli* BL21(DE3), generating the PEB-producing strains PEB7, PEBB, PEB9, PEB6 and PEBP.

The *Nde*I*/Bgl*II fragments of *Ho1* were ligated into *Nde*I*/Bgl*II digested pRSF-*SLA*-*cpcSM7*-*PHo1*-*pebS*, generating the plasmid pRSF-*SLA*-*cpcSM7*-*BHo1*-*pebS*, pRSF-*SLA*-*cpcSM7*-*6Ho1*-*pebS*, pRSF-*SLA*-*cpcSM7*-*7Ho1*-*pebS*, pRSF-*SLA*-cpcSM7-*9Ho1*-*pebS*, respectively. These plasmids were individually transformed into *E. coli* BL21(DE3), generating the strains SLA-V4B, SLA-V46, SLA-V47, SLA-V49, respectively.

*hemL* (from *E. coli*) and *hemA*^*S*^ (a mutant of *hemA* from *Salmonella arizona*, [20]) were designed as a polycistron with a ribosomal binding site engineered upstream of *hemA*. This polycistron was chemically synthesized and ligated into the first expression cassette of the plasmid pCDFDuet-1, generating pCDF-*hemLA*. The expression cassette including T7 promoter, *hemLA* and terminator was then amplified from pCDF-*hemLA* with the primers hemLAF and hemLAR (Additional file 1: Table S1). The PCR product was digested with *Sac*I*/EcoR*I, purified and ligated into the similarly digested plasmid pKIKO*arsB*Cm [21], generating pKIKO*arsB*-*hemLA*. *hemB* was amplified from *E. coli* genomic DNA with the primers hemBF and hemBR (Additional file 1: Table S1). The PCR product was digested with *Nde*I*/Xho*I and ligated into similarly digested pKIKO*arsB*-*hemLA*, generating plasmid pKIKO*arsB*-*hemLAB*. Following a similar procedure, pKIKO*arsB*-*hemLAD*, pKIKO*arsB*-*hemLAF*, pKIKO*arsB*-*hemLAG*, and pKIKO*arsB*-*hemLAH* were constructed. These plasmids were individually electroporated into *E. coli* BL21(DE3) transformed with plasmid pKD46 [22], generating the *E. coli* strains EAL, EALB, EALD, EALF, ELAG and EALH. Integration of the expression cassettes was confirmed by PCR using gene-specific primers and chromosome-specific primers (Additional file 1: Table S1). pRSF-*SLA*-*cpcSM7*-*BHo1*-*pebS* was individually transformed into the strains EAL, EALB, EALD, EALF, ELAG and EALH, generating the *E. coli* strains SLA-V5AL, SLA-V5ALB, SLA-V5ALD, SLA-V5ALF, SLA-V5ALG and SLA-V5ALH, respectively (Fig. 2, Table 1).

**Table 1:** Plasmids used in this study

Plasmids	Description	Source
pRSFDuet-1	Double T7 promoters, RSF ori, Km^R^	Novagen
pCDFDuet-1	Double T7 promoters, P15A ori, Cm^R^	Novagen
pRSF-*PHo1*-*pebS*	pRSFDuet-1 containing *PHo1* and *pebS*	Wu et al. [17]
pCDF-*SLA*-*cpcS*	pCDFDuet-1 containing *SLA* and *cpcS*	Wu et al. [17]
pRSF-*SLA*-*cpcS*-*PHo1*-*pebS*	pRSFDuet-1 containing *SLA* and *apcA*, *cpcS*, *PHo1* and *pebS*	This study
pRSF-*SLA*-*cpcSM7*-*BHo1*-*pebS*	pRSFDuet-1 containing *SLA* and *apcA*, *cpcSM7*, *BHo1* and *pebS*	This study
pRSF-*SLA*-*cpcSM7*-*6Ho1*-*pebS*	pRSFDuet-1 containing *SLA* and *apcA*, *cpcSM7*, 6*Ho1* and *pebS*	This study
pRSF-*SLA*-*cpcSM7*-*7Ho1*-*pebS*	pRSFDuet-1 containing *SLA*, *cpcSM7*, 7*Ho1* and *pebS*	This study
pRSF-*SLA*-*cpcSM7*-*9Ho1*-*pebS*	pRSFDuet-1 containing *SLA*, *cpcSM7*, 9*Ho1* and *pebS*	This study
pRSF-*SLA*-*cpcSM7*-*PHo1*-*pebS*	pRSFDuet-1 containing *SLA*, *cpcSM7*, *PHo1* and *pebS*	This study
pRSF-*PHo1*	pRSFDuet-1 containing *PHo1*	This study
pRSF-*6Ho1*	pRSFDuet-1 containing 6*Ho1*	This study
pRSF-*7Ho1*	pRSFDuet-1 containing *7Ho1*	This study
pRSF-*9Ho1*	pRSFDuet-1 containing *9Ho1*	This study
pRSF-*BHo1*	pRSFDuet-1 containing *BHo1*	This study
pRSF-*6Ho1*-*pebS*	pRSFDuet-1 containing *6Ho1* and *pebS*	This study
pRSF-*7Ho1*-*pebS*	pRSFDuet-1 containing *7Ho1* and *pebS*	This study
pRSF-*9Ho1*-*pebS*	pRSFDuet-1 containing *9Ho1* and *pebS*	This study
pRSF-*BHo1*-*pebS*	pRSFDuet-1 containing *BHo1*and *pebS*	This study
pKD46	repA101ts and oriR101 ParaB *exo*, *bet*, *gam araC bla*	Datsenko and Wanner [22]
pKIKO*arsB*Cm	R6K ori, Cm^R^	Sabri et al. [21]
pKIKO*arsB*-*hemLA*	pKIKO*arsB*Cm containing expression cassette of *hemLA*	This study
pKIKO*arsB*-*hemLAB*	pKIKO*arsB*Cm containing expression cassette of *hemLAB*	This study
pKIKO*arsB*-*hemLAD*	pKIKO*arsB*Cm containing expression cassette of *hemLAD*	This study
pKIKO*arsB*-*hemLAF*	pKIKO*arsB*Cm containing expression cassette of *hemLAF*	This study
pKIKO*arsB*-*hemLAG*	pKIKO*arsB*Cm containing expression cassette of *hemLAG*	This study
pKIKO*arsB*-*hemLAH*	pKIKO*arsB*Cm containing expression cassette of *hemLAH*	This study

### Directed evolution of CpcS

The diversify PCR Random Mutagenesis Kit (TaKaRa, Japan) was used to generate a *cpcS* mutant library. The plasmid pCDF-*SLA*-*cpcS* at a concentration of 5 ng/μL was used as the DNA template, and cpcSMF and cpcSMR were used as the primers (Additional file 1: Table S1). The buffer condition was chosen to obtain a mutagenesis rate of 4.6 mutations per 1000 bp. The cycling conditions were 94 °C for 30 s, 94 °C for 30 s, 68 °C for 1 min (25 cycles), and 68 °C for 1 min. The mutagenized *cpcS* fragment was digested with *Sac*I/*Sal*I, and cloned into similarly digested pRSF-*SLA*-cpcS-*PHo1*-*pebS*. The ligation reaction mixture was then transformed into ultra-competent *E. coli* T1 for the generation of a random mutant library.

*Escherichia coli* BL21(DE3) was transformed with the plasmid library. The transformants were spread on LB agar plates (5 g/L yeast extract, 10 g/L tryptone and 10 g/L NaCl, 10 g/L agar, pH 7.4). The LB agar plates contained 1.0 mM ALA and 1.0 g/L lactose, and were cultivated at 18 °C for 96–120 h in darkness. Positive colonies were selected according to color change. The detailed procedure is described in Additional file 1: Figure S1.

### Analysis of BV and PEB

For the analysis of BV and PEB, the method described by Frankenberg was basically followed [23]. The BV and PEB in *E. coli* cells was extracted with acetone overnight in darkness. The extractions were filtered through 0.45 μm polytetrafluoroethylene syringe filter and then were resolved by reversed-phase chromatography using an Agilent Technologies 1260 system. The HPLC column was a 4.6 × 250-mm Phenomenex Ultracarb analytical column. The mobile phase consisted of acetone and 20 mM formic acid (50:50 by volume), and the flow rate was 0.6 mL/min. The eluates were monitored at 650, 560, and 380 nm using an Agilent Technologies 1260 series diode array detector. As needed, complete spectra were obtained for the peaks desired. Peak areas were quantified using Agilent Technologies Chemstation software.

### Media and culture conditions

LB medium was used for *E. coli* cultivation for the DNA manipulations and seed cultures. For expression of foreign proteins, TB medium was used, which contains 12 g/L tryptone, 24 g/L yeast extract, 4 g/L glycerol, 2.31 g/L KH_2_PO_4_, 12.54 g/L K_2_HPO_4_. Kanamycin (Km, 100 μg/mL) and/or spectinomycin (Sm, 100 μg/mL) were added to culture medium to provide selective pressure. Single colonies of recombinant *E. coli* were grown in 3 mL LB medium. 15 mL of overnight seed cultures were then inoculated into 300 mL of TB medium containing the appropriate antibiotics and cultured at 37 °C until the optical density at 600 nm reached 0.8. The cultures were then cooled to 18 °C, and the expression of the foreign genes was induced by the addition of isopropyl-β-d-thiogalactoside (IPTG) at a final concentration of 0.1 mM. After a further incubation for 24 h at 18 °C in the dark, the *E. coli* cells were harvested by centrifugation at 6000×*g* for 10 min.

The fed-batch fermentation was performed in a 5 L fermentor (BioF6005S-G, China). 2.0% inoculation of overnight seed culture was transferred into the fermentor with 2.5 L TB medium. The constant feeding mode (0.01 g/L/min) was employed to achieve high cell density. The feeding medium was composed of 200 g/L gluocose, 5 g/L NaCl, 5 g/L MgSO_4_·H_2_O, 7 g/L KH_2_PO_4_, 8 g/L K_2_HPO_4_. The culture was cooled to 18 °C when the cells were in the mid-exponential stage. The cells were then induced with 0.5 mM IPTG and cultivated for 18 h.

### Purification of the fusion proteins

The cells were suspended in 30 mL of binding buffer (20 mM sodium phosphate, 500 mM sodium chloride and 20 mM imidazole, pH 7.4) and disrupted by sonication for 30 min on ice. After centrifugation at 6000×*g* for 30 min at 4 °C, the supernatants were loaded onto a pre-equilibrated column of Chelating Sepharose (GE Healthcare BioScience, Sweden) charged with Ni^2+^. The column was then washed with washing buffer (20 mM sodium phosphate, 500 mM sodium chloride and 50–100 mM imidazole, pH 7.4) to remove weakly bound proteins. The fusion proteins were eluted with elution buffer (20 mM sodium phosphate, 500 mM sodium chloride and 500 mM imidazole, pH 7.4). The high-concentration imidazole was removed using a Sephadex G25 column. The peak fraction was collected and concentrated by ultrafiltration. Protein concentrations were measured by the dye-binding method of Bradford using a commercial kit (BestBio, China).

### SDS-PAGE analysis

Expression of Ho1 in *E. coli* was analyzed by SDS-PAGE. *E. coli* cells expressing Ho1 were disrupted by sonication and then centrifugated at 6000×*g* for 30 min at 4 °C. The supernatant was mixed with loading buffer and boiled for 10 min. 10 μL protein samples were loaded on 12% acrylamide gel. Resolved proteins were visualized by staining with Coomassie Blue.

### Spectral analysis

Absorption spectra were obtained using a UV-1801 spectrophotometer (Rayleigh, China) with a 0.5 cm path length cuvette. The wavelength range was from 250 to 700 nm, and the sample interval was set to 1 nm with a scan speed of 240 nm/min.

The fluorescence emission spectra were analyzed using an F-4500 fluorescence spectrophotometer (Hitachi, Japan). The excitation wavelength was 520 nm, and the emission spectra were recorded from 540 to 610 nm. The emission and excitation slit widths were set to 5 nm with a scan speed of 240 nm/min.

The recombinant proteins were denatured in 8.0 M urea (pH 1.5). The PEB concentrations were calculated using an excitation coefficient of 42.8 mM^−1^ cm^−1^ at 560 nm. To calculate the chromophorylation ratio, the concentration of PEB was divided by the concentration of purified recombinant proteins.

### Heme determination

The intracellular heme concentration was determined according to the procedure described by Sassa with slight modification [24, 25]. The pellets were washed once with water, resuspended in 500 μL of 20 mM oxalic acid and then stored in the dark at 4 °C for 16 h. After the acid extraction, 500 μL of 2 M preheated oxalic acid was added. Half of the mixture was transferred to a new amber centrifuge tube and heated to 95 °C for 30 min, during which the other half mixture was kept at room temperature. Two replicate 200 μL aliquots of each sample (heated and unheated) were measured in a microplate reader (Tecan) with excitation at 400 nm and emission measurement at 620 nm. A standard curve was constructed using different concentrations of hemin (Sigma-Aldrich).

### Plasmid stability analysis

The plasmid stability for the strains SLA-V1 and SLA-V2 were compared at the end of expression period. SLA-V1 was grown in TB medium containing 100 μg/mL Sm and 100 μg/mL Km, while SLA-V2 was grown in TB medium containing 100 μg/mL Km. To evaluate the effect of IPTG induction on plasmid stability in these strains, these cultures were induced with or without IPTG at a final concentration of 0.1 mM. One hundred microliter samples were taken from flasks at the end of the 24 h expression period. Each sample was diluted in LB medium and spread on nonselective LB agar plates. These plates were incubated at 37 °C for 16 h. Individual colonies were randomly chosen and replica-plated on various selective LB plates containing 100 μg/mL Sm, 100 μg/mL Km, or 100 μg/mL Sm and 100 μg/mL Km. The plasmid stability was defined as the fraction of plasmid(s)-bearing cells and was determined by taking the ratio of the number of colonies on the selective plates to the total number of transferred colonies.

To determine plasmid structural stability, colony PCR amplification using primers duetup1/duetup2 and T7 terminator was carried out to analyze the stability of expression cassettes.

## Results and discussion

### Increasing the chromophorylation ratio of SLA-PEB by improving plasmid stability

The engineered *E. coli* strain SLA-V1 was constructed in our previous study to produce SLA-PEB. In this strain, the fusion protein (SLA) of core streptavidin from *Streptomyces avidinii* and allophycocyanin alpha subunit from thermophilic cyanobacterium *Thermosynechococcus elongatus* BP-1, together with lyase CpcS from *Thermosynechococcus elongatus* BP-1 and PEB producing enzymes (PHo1 and PebS) from cyanophage PSSM-2, were co-expressed in *E. coli*. Two plasmids for were used for the expression of the four genes required for SLA-PEB biosynthesis [17]. The recombinant SLA-PEB, however, was incompletely chromophorylated, with a chromophorylation ratio of 16.2% (Table 2). This result agreed with previous reports that PBPs were partially chromophorylated when they were expressed in *E. coli* [13, 14, 18]. The underlying mechanisms are not clear at present. Since incomplete chromophorylation leads to low fluorescence emission and detection sensitivity, it is crucial to achieve full chromophorylation of SLA-PEB. So far, most recombinant phycobiliproteins were expressed in *E. coli* using dual or multiple plasmid expression systems [13, 14, 19]. The genes responsible for PBP biosynthesis were expressed using two or more plasmids. Theoretically, the pathway works efficiently only if all of the genes are expressed successfully and in the proper balance. However, the expression plasmid(s) might be lost during the expression of foreign proteins, even in the presence of antibiotic [26–28]. We speculate that plasmid stability in *E. coli* is an important factor affecting the chromophorylation of recombinant SLA-PEB.

**Table 2:** DNA and amino acid changes in the CpcS mutants and chromophorylation ratios of SLA-PEB purified from the wild type and mutant strains

Strains	CpcS mutants	Base changes	Residual changes	Chromophorylation ratio (%)
SLA-V1	CpcS	–	–	16.2 ± 1.2
SLA-V2	CpcS	–	–	38.6 ± 4.6
SLA-V3-2	CpcSM2	T100C, A125G	S34P, E42G	56.2 ± 6.0
SLA-V3-7	CpcSM7	A302G, A460T	E101G, A177P	64.7 ± 3.2
SLA-V3-9	CpcSM9	T30C, A167G, A302G	Q56R, E101G	46.9 ± 5.5
SLA-V3-12	CpcSM12	A287G, C316T, T410C	Q96R, I137T	61.4 ± 5.8
SLA-V3-16	CpcSM16	T127C, T172C, A221G	Y58H, D74G	53.6 ± 2.7

Data represent mean ± SD from three independent experiments

**Fig. 3:**
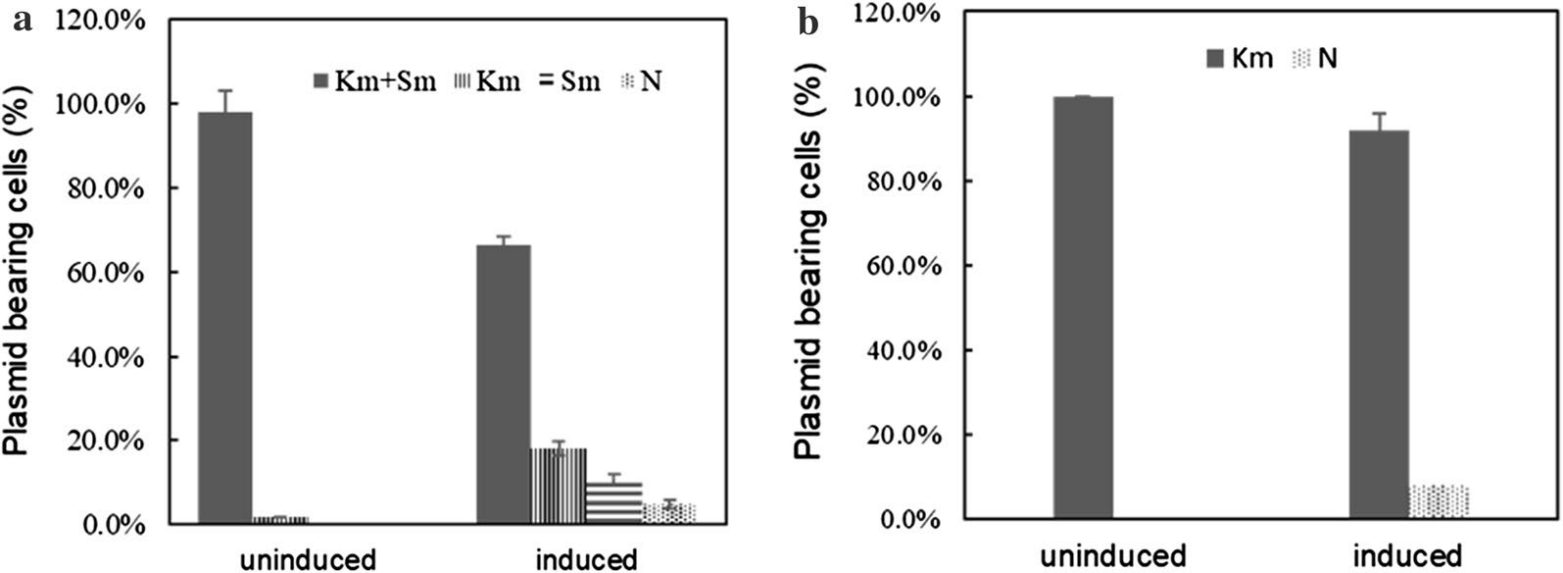
Plasmid stability for the strains SLA-V1 (**a**) and SLA-V2 (**b**). Km + Sm: fraction of *E. coli* cells bearing pCDF-*SLA*-*cpcS* and pRSF-*PHo1*-*pebS*, km: fraction of *E. coli* cells bearing only pRSF-*PHo1*-*pebS* (SLA-V1) or only pRSF-*SLA*-*cpcS*-*PHo1*-*pebS* (SLA-V2), Sm: fraction of *E. coli* cells bearing only pCDF-*SLA*-*cpcS*, N: fraction of plasmid-free *E. coli* cells. Data represent mean ± SD from five independent experiments

The plasmid stability during fermentation was examined for the strain SLA-V1. A fraction of induced *E. coli* cells could not grow on selective plates, indicating plasmid instability in the *E. coli* cells. Under uninduced conditions, the *E. coli* cells had high plasmid stability. Only 1.9% of the *E. coli* cells lost the plasmid pCDF-*SLA*-*cpcS*. Under induced conditions, 16.2% of the *E. coli* cells lost the plasmid pCDF-*SLA*-*cpcS*, 10.6% of the induced *E. coli* cells lost the plasmid pRSF-*PHo1*-*pebS*, and 6.9% of the induced *E. coli* cells lost both plasmids. Only 66.3% of the induced cells retained both expression plasmids (Fig. 3a). These results showed that induction by IPTG led to plasmid loss and heterogenous cell populations. This observation could be confirmed by the results of PCR assays to detect the expression plasmids (Additional file 1: Figure S2). Among 48 randomly selected colonies from the induced cultures, 7 colonies lost pCDF-*SLA*-*cpcS*, 6 colonies lost pRSF-*PHo1*-*pebS*, 4 colonies lost, and 31 colonies retained both plasmids. Due to lack of the PEB biosynthesis pathway, the *E. coli* cells retaining pCDF-*SLA*-*cpcS* could only produce apo-SLA and contributed to the incomplete chromophorylation of recombinant SLA-PEB.

To improve plasmid stability, the biosynthetic pathway for SLA-PEB was reconstructed by using one expression plasmid. The *SLA* and *cpcS* sequences were combined into a polycistron and ligated into the first expression cassette in pRSFDuet-1, while *PHo1* and *pebS* were combined into another polycistron and ligated into the second expression cassette in pRSFDuet-1 (Fig. 1). The resulting plasmid pRSF-*SLA*-*cpcS*-*PHo1*-*pebS* was transformed into *E. coli* BL(DE21), generating the strain SLA-V2. Under uninduced conditions, no *E. coli* cells lost the expression plasmid. Under induced conditions, 5.2% of the *E. coli* cells lost the expression plasmid (Fig. 3b). This result demonstrated an improved plasmid stability in the strain SLA-V2. It should be noted that the plasmid-bearing cells had an entire SLA-PEB biosynthesis pathway, as indicated by PCR amplification of the whole expression cassettes containing the foreign genes (Additional file 1: Figure S3).

SLA-PEB purified from the strain SLA-V2 had a chromophorylation ratio of 38.6%, which was 2.4-fold higher than that purified from SLA-V1 (Table 2). The results confirmed the speculation that plasmid stability is an important factor determining the chromophorylation ratio of recombinant PBP. Considering that both Sm and Km are needed during fermentation of the strain SLA-V1 while only Km is needed for the cultivation of the strain SLA-V2, the cost for production of SLA-PEB in large scale fermentation will be reduced when the strain SLA-V2 is used. From these results, it can be concluded that when a phycobiliprotein biosynthetic pathway was constructed in recombinant *E. coli*, a single-expression plasmid was preferable to two or multiple expression plasmids.

### Molecular evolution of CpcS for the improvement of chromophorylation efficiency

The incomplete chromophorylation of SLA-PEB purified from strain SLA-V2 indicated that there were still factor(s) limiting the chromophorylation of recombinant SLA-PEB. Both PEB depletion and low activity and/or expression level of CpcS would cause incomplete chromophorylation. It was shown by HPLC analysis that PEB accumulated in SLA-V2 cells (Additional file 1: Figure S4A). These data suggested that incomplete chromophorylation of the recombinant PBP was not limited by the availability of PEB. Instead, it was due to low activity and/or expression level of CpcS.

**Fig. 4:**
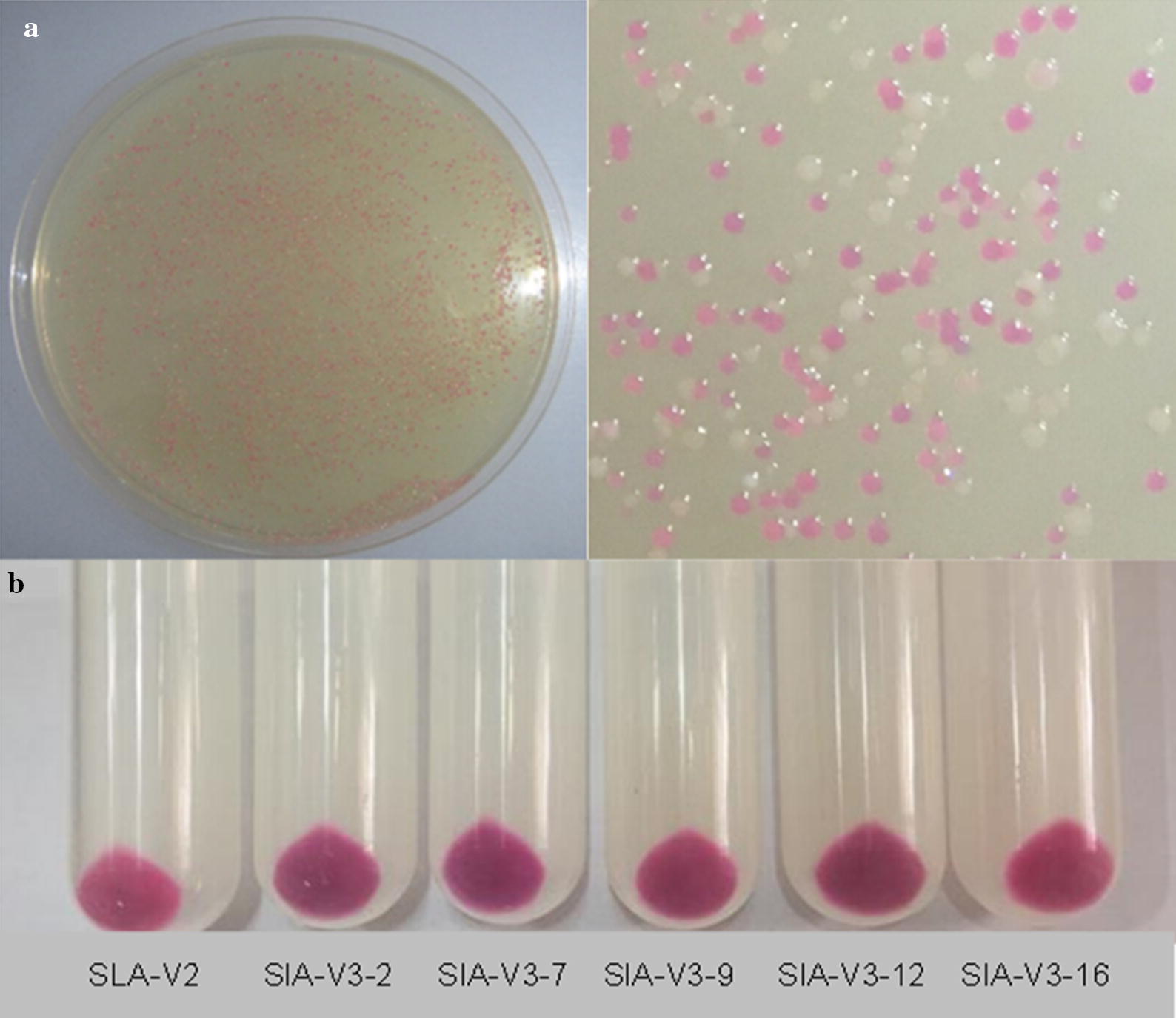
Directed evolution of CpcS. **a** The expression of wild-type CpcS resulted in pink clones and a null mutation in CpcS resulted in white clones, while the clones expressing CpcS mutant with enhanced activities showed red or purple coloration, which could be easily distinguished from the library. **b** Cell pellets of the wild type and five selected mutants

Directed evolution is an efficient strategy for altering the catalytic characteristics of enzymes. Generally, high throughput screening of rare but desirable clones from a mutant library is the most critical step in laboratory directed evolution [29]. To improve the catalytic performance of CpcS, a directed evolution strategy was developed based on agar plate screening (Fig. 4). The wild type clones appeared pink on agar plates, while the clones expressing CpcS mutants with increased activity had a deep color. A mutant library of approximately 5 × 10^4^ clones was created by random mutagenesis using error-prone PCR. The insertion rate of the library was 92.6%, and the average mutation rate was 5.3 mutations per 1000 bp, as estimated by DNA sequencing of 48 randomly selected colonies. Seventeen colonies were selected from the mutant library based on color change. To examine whether these strains indeed had enhanced chromophorylation ability, recombinant SLA-PEB was purified from these strains. Five strains were confirmed to be authentic positive mutants with increased chromophorylation ratios (Table 2). DNA sequencing revealed that the mutants encoded single or multiple amino acid substitutions. The enhanced performance for SLA-PEB chromophorylation might result from enhanced affinity for PEB and higher catalytic efficiency of the CpcS mutant. Among these five colonies, the strain SLA-V3-7 (denoted as SLA-V3) expressing CpcSM7 had the best performance, producing SLA-PEB with a chromophorylation ratio of 64.7%.

### Phycobilin biosynthesis using Ho1 from different cyanobacteria

Although the chromophorylation ratio of SLA-PEB in strain SLA-V3 was elevated significantly, there was still a fraction of SLA-PEB in its apo-form. PEB biosynthesis in the recombinant *E. coli* cells was achieved via expression of *PHo1* and *pebS*. Endogenous heme served as the precursor for PEB biosynthesis. The conversion of heme to biliverdin IX was catalyzed by the heme oxygenase, and then the conversion of biliverdin IX to PEB was catalyzed by the ferredoxin-dependent bilin reductase pebS (R). HPLC analysis showed that in SLA-V3 cells neither PEB nor BV could be detected (Additional file 1: Figure S4B). We speculated that in strain SLA-V3, the chromophorylation of recombinant SLA-PEB is limited by low Ho1 activity and/or heme depletion.

**Fig. 5:**
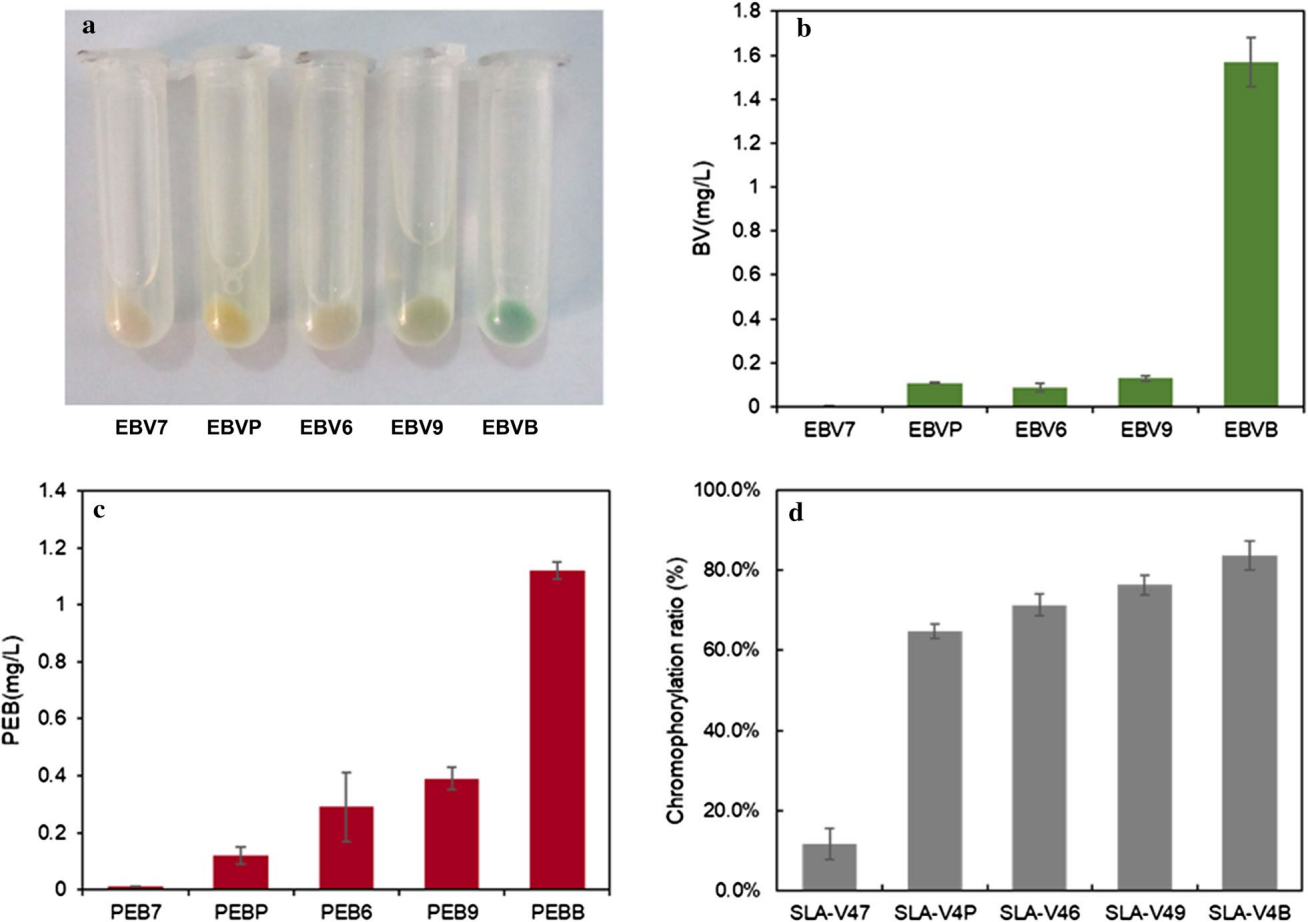
Effects of different Ho1s on the production of BV and PEB and chromophorylation of SLA-PEB. **a** Cell pellets for the strains expressing Ho1s from different cyanobacteria. **b** BV production for the strains expressing Ho1s from different cyanobacteria. **c** PEB production for the strains coexpressing pebS and Ho1s from different cyanobacteria. **d** Chromophorylation ratios of SLA-PEB purified from the strains coexpressing pebS and Ho1s from different cyanobacteria. Data represent mean ± SD from three independent experiments

Here, the directed evolution strategy was also employed, with the aim to increase the catalytic activity of PHo1. Unfortunately, no positive mutant was obtained from the screen. Alternatively, we tried to identify Ho1 homologs with high activity and/or expression levels from different cyanobacteria. Genes coding putative Ho1 can be found in the genome of sequenced cyanobacteria. Four Ho1s from cyanobacteria with diverse growth conditions were chosen as candidates. The marine cyanobacterium *Synechococcus* sp. PCC 7002 and freshwater cyanobacterium *Synechococcus elongatus* BP-1 can grow at high temperature [30, 31], while the marine cyanobacterium *Synechococcus* sp. PCC 9311 and freshwater cyanobacterium *Synechocystis* sp. PCC 6803 grow at room temperature. All *Ho1*s were codon optimized and expressed in *E. coli*. The catalytic abilities of these Ho1s to convert heme to BV in *E. coli* were compared. The *E. coli* strain expressing *BHo1* turned green after 24 h of induction with IPTG, while *E. coli* strains expressing the other *Ho1*s did not show obvious color changes (Fig. 5). HPLC analysis showed that among the five BV-producing strains, the strain EBVB had the best performance in BV accumulation, producing 14.3-fold higher BV than the strain BVP. Similarly, the PEB-producing strain PEBB accumulated the highest level of PEB, which was 9.3-fold higher than the strain PEBP. It appeared that the high catalytic ability of BHo1 was attributed to its high expression levels in *E. coli*, since SDS-PAGE showed that BHo1 was expressed at the highest level among the five Ho1s (Additional file 1: Figure S5).

To examine whether improved PEB accumulation would promote the chromophorylation of recombinant proteins in *E. coli*, *PHo1* in strain SLA-V3 was replaced by different *Ho1*s (Fig. 1). A positive correlation between PEB accumulation and chromophorylation ratio of SLA-PEB was observed (Fig. 5). SLA-PEB purified from the strain SLA-V4B had a chromophorylation ratio of 83.6%, compared to 64.7% for protein purified from the strain SLA-V3. These results confirmed that in strain SLA-V3, PHo1 was a limiting factor for efficient chromophorylation of HT-SLA.

### Upregulation of the heme biosynthetic pathway genes for further improvement of chromophorylation efficiency

Heme is an essential molecule for cells due to its involvement in many important processes [32]. In engineered microorganisms that express heme proteins, excessive heme consumption disrupts the host metabolism and limits the production of heme proteins [33, 34]. An increased heme level resulted in a significant enhancement of human hemoglobin production [35]. In this study, endogenous heme served as the precursor for PEB biosynthesis. No free PEB could be detected in the strain SLA-V4B (Additional file 1: Figure S4C), suggesting that heme might be a limiting factor for full chromophorylation of SLA. Increased heme availability would presumably improve chromophorylation efficiency of the recombinant protein. However, the addition of heme is not suitable in large-scale production processes due to its high hydrophobicity and cost. Instead, regulation of the heme pathway by metabolic engineering is preferable.

HemL and HemA are the rate-limiting enzymes in the heme biosynthesis pathway in *E. coli* [36, 37]. In this study, *hemL* and *hemA* were combined into an operon and inserted into the chromosomal *ArsB* locus of *E. coli* through allelic exchange, generating the strain ELA. Upregulation of *hemA* and *hemL* significantly increased heme accumulation (Table 3), demonstrating a critical role of *hemA* and *hemL* in heme metabolism. In addition to *hemL* and *hemA*, *hemB*, *hemD*, *hemF*, *hemG* and *hemH* were reported to be the regulatory targets of heme biosynthesis pathway in *E. coli*. Overexpression of these genes affected heme biosynthesis pathway [38]. To further improve heme biosynthesis, each of these genes was combined into a polycistron together with *hemL* and *hemA* and integrated into the chromosomal *arsB* locus in *E. coli* (Table 3). Compared to strain V5AL, strain V5ALD produced more heme, while the strains V5ALB, V5ALF, V5ALG and V5ALH produced equivalent or reduced heme levels. SLA-PEB purified from strain V5ALD showed a chromophorylation ratio of 98.6%. This was the highest chromophorylation ratio reported to date.

**Table 3:** Effects of the overexpression of heme biosynthetic genes on heme accumulation and chromophorylation ratio of SLA-PEB

*E. coli* strain	Expressed heme gene(s)	Heme (μmol/L)	Chromophorylation ratio (%)
SLA-V4B	–	1.94 ± 0.16	83.6 ± 4.92
V5AL	*hemA*, *hemL*	2.96 ± 0.26	94.7 ± 3.23
V5ALB	*hemA*, *hemL*, *hemB*	1.95 ± 0.17	85.3 ± 2.15
V5ALD	*hemA*, *hemL*, *hemD*	3.30 ± 0.24	98.6 ± 3.98
V5ALF	*hemA*, *hemL*, *hemF*	2.78 ± 0.44	89.3 ± 3.53
V5ALG	*hemA*, *hemL*, *hemG*	2.36 ± 0.18	87.4 ± 2.70
V5ALH	*hemA*, *hemL*, *hemH*	1.70 ± 0.19	85.2 ± 4.12

Data represent mean ± SD from five independent experiments

### SLA-PEB production in fed-batch fermentation

**Fig. 6:**
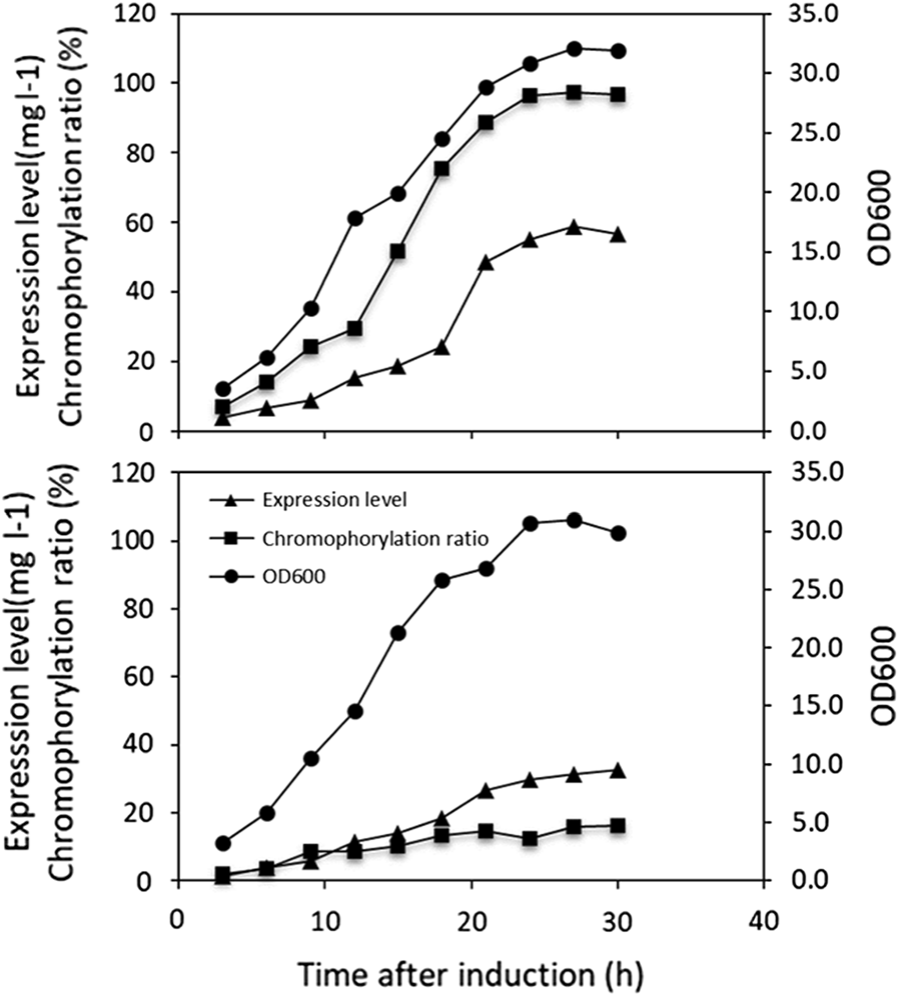
Time course of OD_600_, chromophorylation ratio and production level of SLA-PEB in fed-batch fermentation for the strains SLA-V1 (**a**) and SLA-V5ALD (**b**)

We noticed that when *E. coli* strain producing PBPs were cultured on large-scale (e.g., in a fermenter), the chromophorylation ratio of recombinant PBPs was remarkably decreased, though its production level was elevated (data not shown). To examine whether the strain V5ALD had superior performance in PBP production, both *E. coli* strains were cultivated in a 5 L fermentor. IPTG was added to the culture medium at a final concentration of 1.0 mM when the cell density reached an OD_600_ of 8.0. Constant feeding mode was employed to achieved high cell density. While both strains had similar cell densities at the end of the fermentation, strain V5ALD produced a higher level of SLA-PEB (56.4 mg/L) than the strain SLA-V1 (32.5 mg/L). Most importantly, the chromophorylation ratio of the SLA-PEB purified from V5ALD was 96.7%, which was much higher than that from strain SLA-V1 (Fig. 6).

## Conclusion

An ideal cell factory should demonstrate high efficiency of target product biosynthesis. Inefficient biosynthesis of recombinant PBPs in recombinant *E. coli* leads to the production of a mixture of holo-PBP and apo-PBP. In this study, the biosynthesis pathway for phycobiliprotein was constructed using one expression plasmid, which had significantly increased plasmid stability. Moreover, strategies for the directed evolution of lyase, the upregulation of heme biosynthetic pathway genes and for overcoming the limiting factors in PEB biosynthesis were employed to enhance the production level and chromophorylation of recombinant phycobiliprotein in *E. coli*. A chromophorylation ratio of 96.7% for SLA-PEB was achieved in fed-batch fermentation in this study, and this value was the highest ever reported from an engineered *E. coli* strain. This study demonstrates the validity of a single-expression vector strategy and metabolic engineering for establishing an efficient biosynthetic pathway for PBP production in *E. coli*.

## Additional file

**Additional file 1: Table S1.** Primers used in this study. **Table S2.** Recombinant *E. coli* strains used in this study. **Figure S1.** Flowchart of directed evolution of CpcS for improving chromophorylation efficiency of SLA-PEB. **Figure S2.** Colony PCR for detection of the presence of pCDF-SLA-cpcS and pRSF-PHo1-pebS in *E. coli* strain SLA-V1. The primers are duetup-2 and T7 terminator. The expression cassette of cpcS in pCDF-SLA-cpcS is 813 bp, and the expression cassette of Ho1 and pebS is 1709 bp. DNA marker: 8000 bp, 5000 bp, 3000 bp, 2000 bp, 1000 bp, 750 bp, 500 bp, 250 bp and 100 bp. **Figure S3.** Colony PCR for detection of the stability of expression cassette. The primers are duetup-1 and T7 terminator. The size of expression cassette of SLA, CpcS, PHo1 and pebS is 3433 bp. DNA marker: 8000 bp, 5000 bp, 3000 bp, 2000 bp, 1000 bp, 750 bp, 500 bp, 250 bp and 100 bp. **Figure S4.** HPLC analysis of PEB extracted from *E. coli* strains. A: SLA-V2; B: SLA-V3; C: SLA-V4B. **Figure S5.** SDS-PAGE analysis of Ho1s expression in *E. coli* strains. 1: EBV7, 2: EBVP, 3: EBV6, 4: EBV9, 5: EBVB. The calculated molecular weight is 27.1 kDa for 7Ho1, 26.9 kDa for PHo1, 27.0 kDa for 6Ho1, 26.7 kDa for 9Ho1 and 27.1 kDa for BHo1.
