# Supplementary material for: Metabolic engineering of Escherichia coli for efficient biosynthesis of fluorescent phycobiliprotein
Source: Microb Cell Fact. 2019 Mar 20;18:58. doi: 10.1186/s12934-019-1100-6 (PMC6425641; doi:10.1186/s12934-019-1100-6)
Supplement: Supplementary file 1 — Additional file 1: Table S1. Primers used in this study. Table S2. Recombinant E. coli strains used in this study. Figure S1. Flowchart of directed evolution of CpcS for improving chromophorylation efficiency of SLA-PEB. Figure S2. Colony PCR for detection of the presence of pCDF-SLA-cpcS and pRSF-PHo1-pebS in E. coli strain SLA-V1. The primers are duetup-2 and T7 terminator. The expression cassette of cpcS in pCDF-SLA-cpcS is 813 bp, and the expression cassette of Ho1 and pebS is 1709 bp. DNA marker: 8000 bp, 5000 bp, 3000 bp, 2000 bp, 1000 bp, 750 bp, 500 bp, 250 bp and 100 bp. Figure S3. Colony PCR for detection of the stability of expression cassette. The primers are duetup-1 and T7 terminator. The size of expression cassette of SLA, CpcS, PHo1 and pebS is 3433 bp. DNA marker: 8000 bp, 5000 bp, 3000 bp, 2000 bp, 1000 bp, 750 bp, 500 bp, 250 bp and 100 bp. Figure S4. HPLC analysis of PEB extracted from E. coli strains. A: SLA-V2; B: SLA-V3; C: SLA-V4B. Figure S5. SDS-PAGE analysis of Ho1s expression in E. coli strains. 1: EBV7, 2: EBVP, 3: EBV6, 4: EBV9, 5: EBVB. The calculated molecular weight is 27.1 kDa for 7Ho1, 26.9 kDa for PHo1, 27.0 kDa for 6Ho1, 26.7 kDa for 9Ho1 and 27.1 kDa for BHo1. [file 12934_2019_1100_MOESM1_ESM.docx]

| Primers | Sequence (5’→3’ ) |
| --- | --- |
| pebSF | TCGAAGATCTCATGACGAAGAACCCGCGT |
| pebSR | TCGGGGTACCATTATTTATAGGAAAACAGG |
| hemBF | CGCCATATGACAGACTTAATCCAACGCCC |
| hemBR | CCGCTCGAGATCAGATCTTTAACGCAGAATCTTCTTCTCAGC |
| hemDF | CGCCATATGAGTATCCTTGTCACCCGCC |
| hemDR | CCGCTCGAGATCAGATCTTTATTGTAATGCCCGTAAAAGCG |
| hemFF | CGCCATATGAAACCCGACGCACACC |
| hemFR | CCGCTCGAGATCAGATCTTTACACCCAATCCCTGACCTTAAT |
| hemGF | CGCCATATGAAAACATTAATTCTTTTCTCAACA |
| hemGR | CCGCTCGAGATCAGATCTTTATTTCAGCGTCGGTTTGTC |
| hemHF | CGCCATATGCGTCAGACTAAAACCGGTATC |
| hemHR | CCGCTCGAGATCAGATCTTTAGCGATACGCGGCAACA |
| hemLAF | tctgaattctgcgccattcgatggtgtcc |
| hemLAR | CTAGAGCTCctcaaatgcctgaggtttca |
| GSP | CAACCTGGCTCGACAAAACT |
| cpcSF | ATAGAGCTCAAGGAGATATACCATGTGCATAG GTATGGAC |
| cpcSR | ATAGTCGACTCAGGAGTTGGCGGGTTGC |
| cpcSMF | GAGCCTATGGAAAAACGCCAGCAACGAATTCAAGGAGATATACCATGTGCATAG GTATGGAC |
| cpcSMR | GCAAAAAAGGGAATAAGGGCGACACGAGCTCTCAGGAGTTGGCGGGTTGC |
| MutFor | GAGCCTATGGAAAAACGCCAGCAAC |
| MutRev | GCAAAAAAGGGAATAAGGGCGACAC |
| Duetup1 | GATCTCGACGCTCTCCCT |
| Duetup2 | TTGTACACGGCCGCATAATC |
| T7 Terminator | GCTAGTTATTGCTCAGCGG |

Table S1 Primers used in this study

| Strains | Description | Source |
| --- | --- | --- |
| SLA-V1 | *E. coli* BL21(DE3) harboring pCDF-*SLA-cpcS*, pRSF-*PHo1-pebS* | Wu et al., 2017 |
| SLA-V2 | *E. coli* BL21(DE3) harboring pRSF*-SLA-cpcS-PHo1-pebS* | This study |
| SLA-V3 | *E. coli* BL21(DE3) harboring pRSF*-SLA-cpcSM7-PHo1-pebS* | This study |
| SLA-V4B | *E. coli* BL21(DE3) harboring pRSF-*SLA-cpcSM7-BHo1-pebS* | This study |
| SLA-V46 | *E. coli* BL21(DE3) harboring pRSF-*SLA-cpcSM7-6Ho1-pebS* | This study |
| SLA-V47 | *E. coli* BL21(DE3) harboring pRSF-*SLA-cpcSM7-7Ho1-pebS* | This study |
| SLA-V49 | *E. coli* BL21(DE3) harboring pRSF-*SLA-cpcSM7-9Ho1-pebS* | This study |
| EBVP | *E. coli* BL21(DE3) harboring pRSF*-PHo1* | This study |
| EBVB | *E. coli* BL21(DE3) harboring pRSF-*BHo1* | This study |
| EBV6 | *E. coli* BL21(DE3) harboring pRSF-*6Ho1* | This study |
| EBV7 | *E. coli* BL21(DE3) harboring pRSF-*7Ho1* | This study |
| EBV9 | E. coli BL21(DE3) harboring pRSF-*9Ho1* | This study |
| PEBP | *E. coli* BL21(DE3) harboring pRSF-*PHo1-pebS* | This study |
| PEBB | E. coli BL21(DE3) harboring pRSF*-BHo1-pebS* | This study |
| PEB6 | *E. coli* BL21(DE3) harboring pRSF*-6Ho1-pebS* | This study |
| PEB7 | *E. coli* BL21(DE3) harboring pRSF*-7Ho1-pebS* | This study |
| PEB9 | *E. coli* BL21(DE3) harboring pRSF*-9Ho1-pebS* | This study |
| EAL | *E. coli* BL21(DE3) *ΔarsB::hemA-hemL* | This study |
| EALB | *E. coli* BL21(DE3) *ΔarsB::hemA-hemL-hemB* | This study |
| EALD | *E. coli* BL21(DE3) *ΔarsB::hemA-hemL-hemD* | This study |
| EALF | *E. coli* BL21(DE3) *ΔarsB::hemA-hemL-hemF* | This study |
| EALG | *E. coli* BL21(DE3) *ΔarsB::hemA-hemL-hemG* | This study |
| EALH | *E. coli* BL21(DE3) *ΔarsB::hemA-hemL-hemH* | This study |
| SLA-V5AL | EAL harboring pRSF-*SLA-cpcSM7-BHo1-pebS* | This study |
| SLA-V5ALB | EALB harboring pRSF-*SLA-cpcSM7-BHo1-pebS* | This study |
| SLA-V5ALD | EALD harboring pRSF-*SLA-cpcSM7-BHo1-pebS* | This study |
| SLA-V5ALF | EALF harboring pRSF-*SLA-cpcSM7-BHo1-pebS* | This study |
| SLA-V5ALG | EALG harboring pRSF-*SLA-cpcSM7-BHo1-pebS* | This study |
| SLA-V5ALH | EALH harboring pRSF-*SLA-cpcSM7-BHo1-pebS* | This study |

Table S2 Recombinant *E. coli* strains used in this study.

Fig. S1 Flowchart of directed evolution of CpcS for improving chromophorylation efficiency of SLA-PEB.


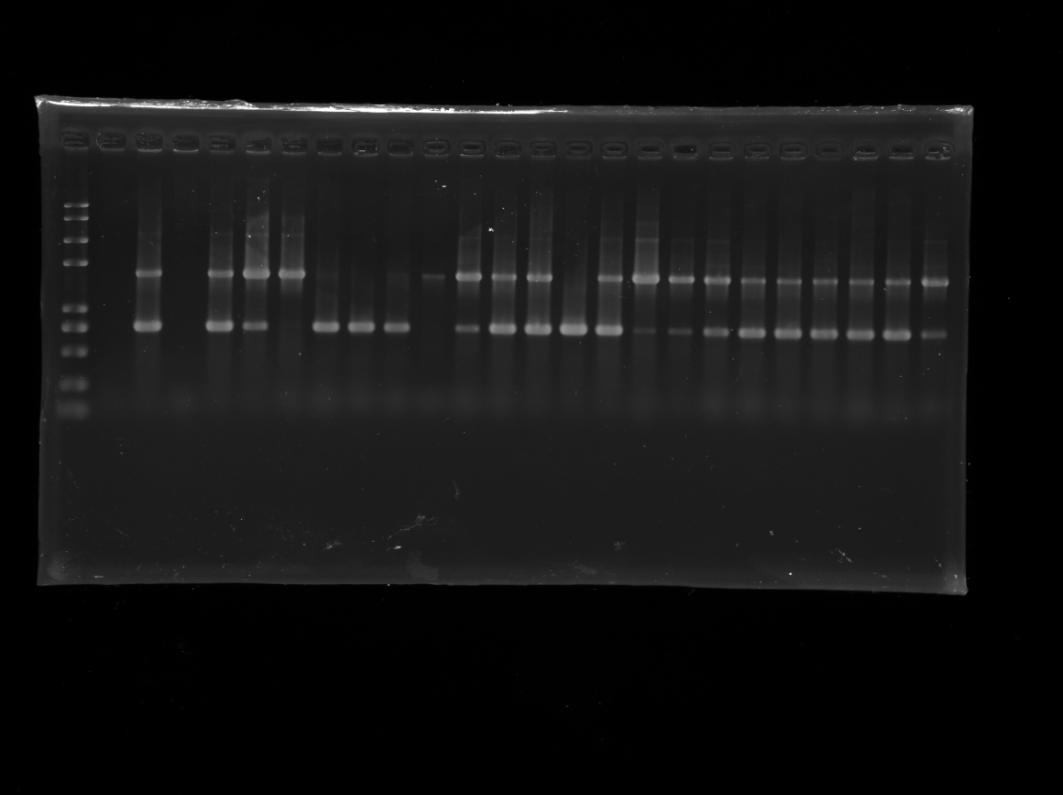

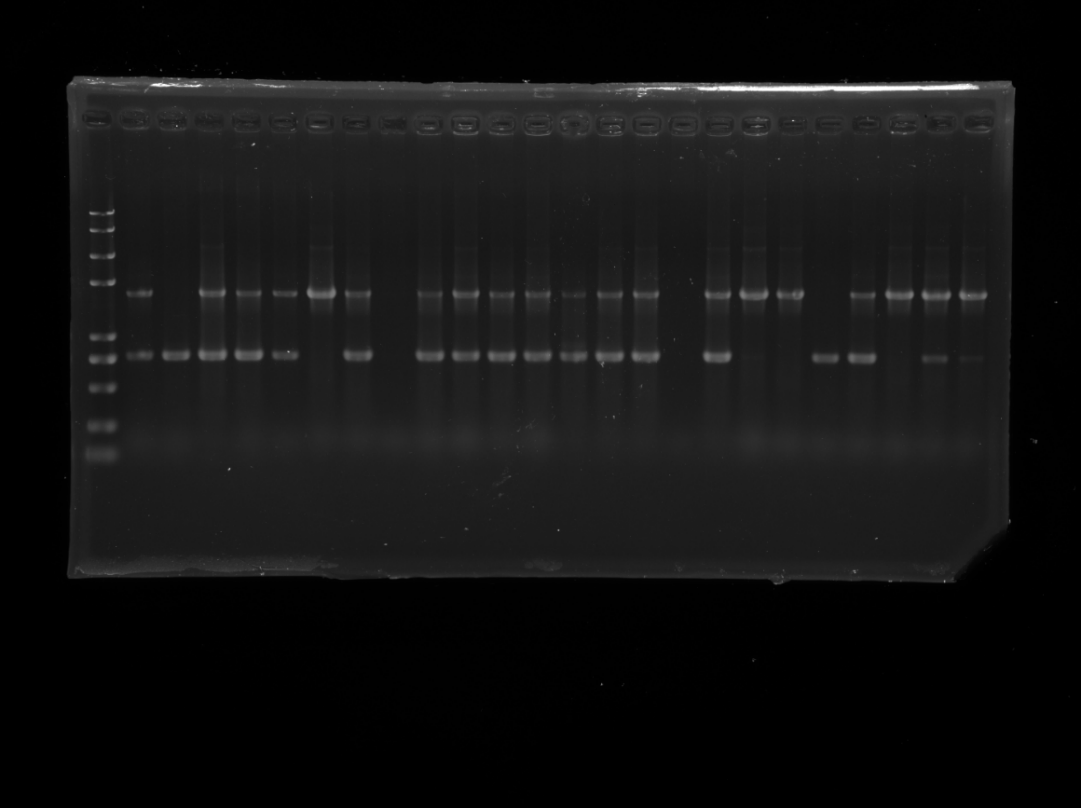


Fig. S2 Colony PCR for detection of the presence of pCDF-SLA-cpcS and pRSF-PHo1-pebS in E. coli strain SLA-V1. The primers are duetup-2 and T7 terminator. The expression cassette of cpcS in pCDF-SLA-cpcS is 813 bp, and the expression cassette of Ho1 and pebS is 1709 bp. DNA marker: 8000 bp, 5000 bp, 3000 bp, 2000 bp, 1000 bp, 750 bp, 500 bp, 250 bp and 100 bp.


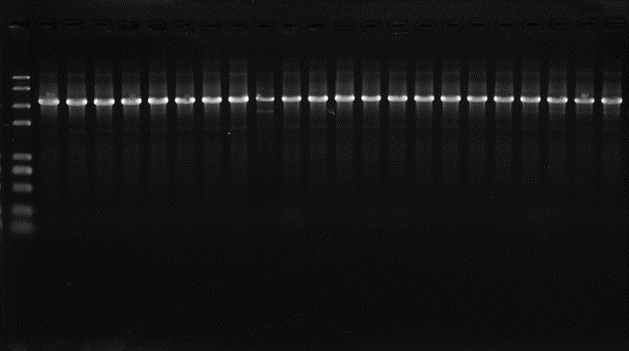


Fig. S3 Colony PCR for detection of the stability of expression cassette. The primers are duetup-1 and T7 terminator. The size of expression cassette of SLA, CpcS, PHo1 and pebS is 3433 bp. DNA marker: 8000 bp, 5000 bp, 3000 bp, 2000 bp, 1000 bp, 750 bp, 500 bp, 250 bp and 100 bp.

Fig. S4 HPLC analysis of PEB extracted from *E. coli* strains. A: SLA-V2; B: SLA-V3; C: SLA-V4B.

Fig. S5 SDS-PAGE analysis of Ho1s expression in *E. coli* strains. 1: EBV7, 2: EBVP, 3: EBV6, 4: EBV9, 5: EBVB. The calculated molecular weight is 27.1 kDa for 7Ho1, 26.9 kDa for PHo1, 27.0 kDa for 6Ho1, 26.7 kDa for 9Ho1 and 27.1 kDa for BHo1.
